# Mice with a deficiency in Peroxisomal Membrane Protein 4 (PXMP4) display mild changes in hepatic lipid metabolism

**DOI:** 10.1038/s41598-022-06479-y

**Published:** 2022-02-15

**Authors:** Maaike Blankestijn, Vincent W. Bloks, Dicky Struik, Nicolette Huijkman, Niels Kloosterhuis, Justina C. Wolters, Ronald J. A. Wanders, Frédéric M. Vaz, Markus Islinger, Folkert Kuipers, Bart van de Sluis, Albert K. Groen, Henkjan J. Verkade, Johan W. Jonker

**Affiliations:** 1 Department of Pediatrics, University of Groningen, University Medical Center Groningen, Groningen, The Netherlands; 2 iPSC/CRISPR Center Groningen, University of Groningen, University Medical Center Groningen, Groningen, The Netherlands; 3 Laboratory of Genetic Metabolic Diseases, Department of Clinical Chemistry, Amsterdam UMC, University of Amsterdam, Amsterdam Gastroenterology Endocrinology Metabolism, Amsterdam, The Netherlands; 4 Department of Pediatrics, Emma Children’s Hospital, Amsterdam UMC, University of Amsterdam, Amsterdam, The Netherlands; 5 Core Facility Metabolomics, Amsterdam UMC, University of Amsterdam, Amsterdam, The Netherlands; 6 Institute of Neuroanatomy, Mannheim Center for Translational Neuroscience, Medical Faculty Mannheim, University of Heidelberg, Mannheim, Germany; 7 Laboratory of Experimental Vascular Medicine, University of Amsterdam, Academic Medical Center, Amsterdam, The Netherlands

**Keywords:** Peroxisomes, Gene regulation

## Abstract

Peroxisomes play an important role in the metabolism of a variety of biomolecules, including lipids and bile acids. Peroxisomal Membrane Protein 4 (PXMP4) is a ubiquitously expressed peroxisomal membrane protein that is transcriptionally regulated by peroxisome proliferator-activated receptor α (PPARα), but its function is still unknown. To investigate the physiological function of PXMP4, we generated a *Pxmp4* knockout (*Pxmp4*^*−/−*^) mouse model using CRISPR/Cas9-mediated gene editing. Peroxisome function was studied under standard chow-fed conditions and after stimulation of peroxisomal activity using the PPARα ligand fenofibrate or by using phytol, a metabolite of chlorophyll that undergoes peroxisomal oxidation. *Pxmp4*^*−/−*^ mice were viable, fertile, and displayed no changes in peroxisome numbers or morphology under standard conditions. Also, no differences were observed in the plasma levels of products from major peroxisomal pathways, including very long-chain fatty acids (VLCFAs), bile acids (BAs), and BA intermediates di- and trihydroxycholestanoic acid. Although elevated levels of the phytol metabolites phytanic and pristanic acid in *Pxmp4*^*−/−*^ mice pointed towards an impairment in peroxisomal α-oxidation capacity, treatment of *Pxmp4*^*−/−*^ mice with a phytol-enriched diet did not further increase phytanic/pristanic acid levels. Finally, lipidomic analysis revealed that loss of Pxmp4 decreased hepatic levels of the alkyldiacylglycerol class of neutral ether lipids, particularly those containing polyunsaturated fatty acids. Together, our data show that while PXMP4 is not critical for overall peroxisome function under the conditions tested, it may have a role in the metabolism of (ether)lipids.

## Introduction

Peroxisomes play important roles in cellular detoxification but also in energy metabolism through oxidation and biosynthesis of lipids and bile acids^1,2^. The importance of peroxisomes is highlighted by diseases in which peroxisomes are malfunctioning either due to a single peroxisomal enzyme deficiency or a defect in peroxisome biosynthesis^3,4^. Mutations in genes encoding peroxisomal proteins lead to a diverse spectrum of disorders, which can be divided into peroxisomal biogenesis disorders and disorders of peroxisome function. The latter group can be further subdivided into enzymatic deficiencies and transport deficiencies^1,4,5^. Peroxisomal disorders are characterized by a broad range of symptoms, which depend on the specific biological pathways controlled by the defective peroxisomal protein. Therefore, symptoms can vary from minimal clinical abnormalities in some patients to severe neurological features and early death depending upon the extent of the deficiency and the identity of the defect involved^6^.

The functions of peroxisomes have been studied by manipulation of genes coding for peroxisomal proteins and by pharmacological manipulation of peroxisomal activity, such as peroxisome proliferators^7–10^. Peroxisome proliferators act through activation of the peroxisome proliferator-activated receptors (PPARs), a subfamily of nuclear receptors^11,12^, thereby stimulating specific peroxisome functions^13,14^. Because PPARs can affect a plethora of metabolic processes, their potential role in the treatment of metabolic disorders has become subject to extensive research^15,16^.

PPARα is a master regulator of lipid metabolism and is highly expressed in tissues with a high rate of fatty acid oxidation, including the liver, brown adipose tissue, (skeletal) muscle, and heart^17^. Endogenous ligands for PPARα include polyunsaturated fatty acids and metabolites such as eicosanoids and leukotriene B4. PPARα plays an important role in the fasting response. Upon activation, PPARα increases fatty acid oxidation and ketogenesis in various tissues such as the liver and muscle and regulates glucose metabolism in the liver^18–22^. In addition, PPARα is the main target of the fibrate class of lipid-lowering drugs that have been clinically used since the 1930s^11^.

Many different genes encoding peroxisomal proteins have been identified over the last few years; however, several of the proteins involved have not yet been characterized. Knowledge about their function could be instrumental for the understanding of the pathogenesis of peroxisomal disorders. By analyzing transcriptome data using the Genevestigator database (genevestigator.com), we identified Peroxisomal Membrane Protein 4 (*Pmp24, Pxmp4*) as a strong target of PPARα. Similarly, Rakhshandehroo et al. (2009) reported the upregulation of *Pxmp4* by the PPARα agonist Wy14643 in human and mouse primary hepatocytes^23–26^. PXMP4 is an integral membrane protein of 212 amino acids and has a molecular mass of 24 kDa^27^. The *PXMP4* gene is ubiquitously expressed and displays strong species conservation. Although various somatic mutations^28^ and hypermethylation resulting in the silencing of PXMP4 in humans have been reported for several types of cancer^29^, its role in tumor development as well as its physiological functions have remained unknown^27^. In the current study, we aimed to address the metabolic function of PXMP4, using mice with a genetic deficiency in PXMP4 in combination with pharmacological approaches to stimulate peroxisomal activity.

## Results

### Generation and characterization of *Pxmp4* knockout mice

**Figure 1:**
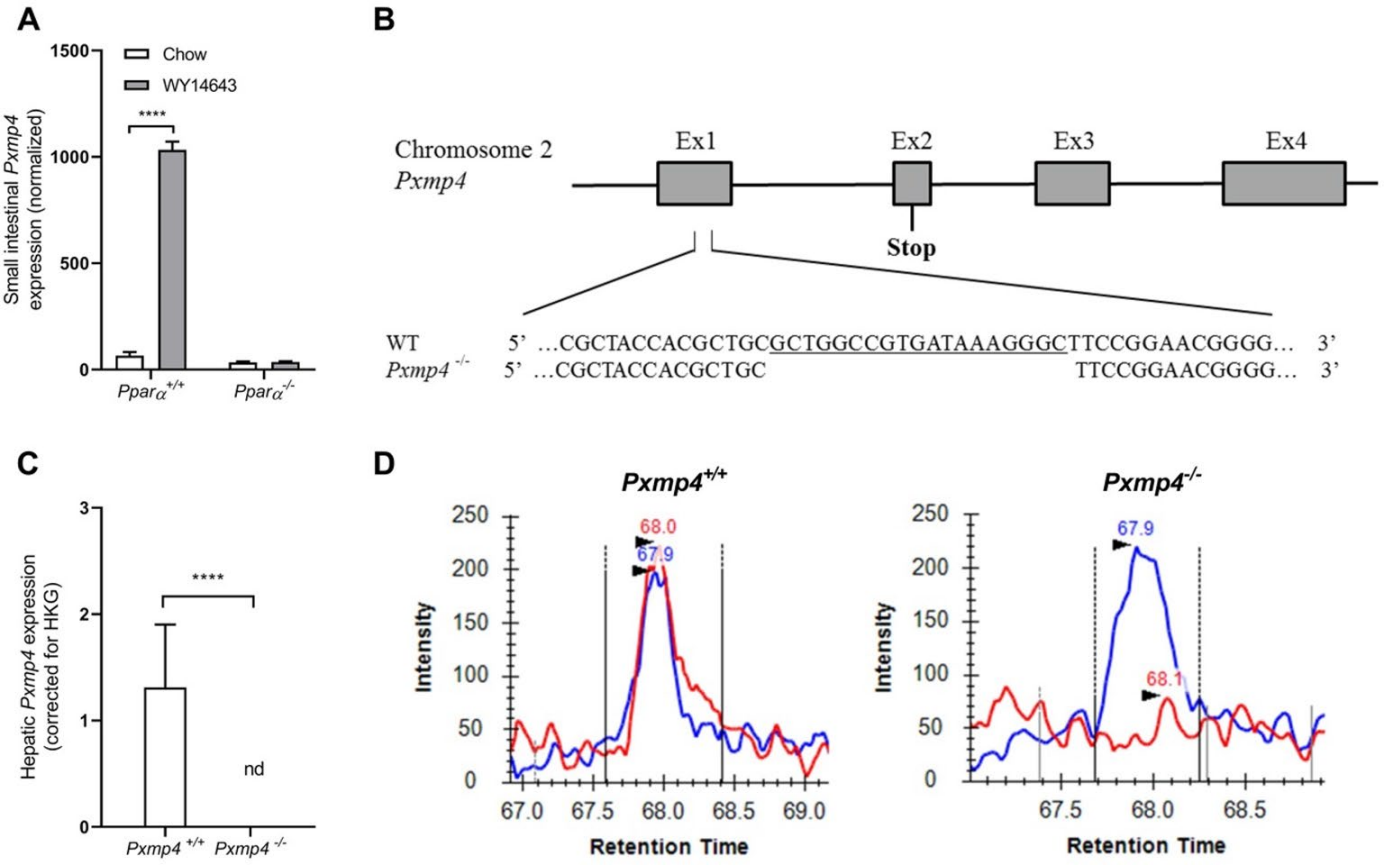
Generation and validation of PXMP4 knockout (*Pxmp4*^*−/−*^) mice. (**A**) Small intestinal *Pxmp4* levels in wild type (*Pparα*^+/+^) and *Pparα*-deficient (*Pparα*^*−/−*^) mice; (**B**) Schematic representation of the *Pxmp4* gene. A 19 bp deletion in exon 1 was introduced by targeted CRISPR/Cas9 gene editing resulting in a premature stop codon in exon 2; (**C**) Hepatic mRNA levels of *Pxmp4*; (**D**) Targeted proteomic analysis of PXMP4 protein in livers of *Pxmp4*^*−/−*^ mice and wild type littermates (n = 5–6). The blue line represents the internal standard and the red line the endogenous levels of PXMP4. Data in figure (**A**) and (**C**) are represented as mean ± SD and statistical significance was tested by a t-test.

By analyzing transcriptome data using the Genevestigator database (genevestigator.com), we identified PXMP4 as a strong target of PPARα (Fig. 1A). The mouse *Pxmp4* gene is located on chromosome 2 and contains four exons, encoding a protein of 212 amino acids (Fig. 1B). A 19 base pair (bp) deletion in exon 1 was introduced by targeted CRISPR/Cas9-mediated gene editing resulting in germline inactivation of *Pxmp4*. This mutation causes a premature stop codon in exon 2, resulting in a loss-of-function protein. Levels of *Pxmp4* mRNA were not detectable in livers of *Pxmp4*^*−/−*^ mice (Fig. 1C). In line with this, endogenous PXMP4 protein levels, as determined by targeted proteomics, were below the detection limit in livers of *Pxmp4*^*−/−*^ mice, whereas PXMP4 was readily detectable in livers of wild type mice (Fig. 1D). *Pxmp4*^*−/−*^ mice were born according to the expected Mendelian ratio, were fertile, and showed no growth retardation or other visible abnormalities (data not shown). Under standard chow conditions, no differences were observed in body weight and fat mass between *Pxmp4*^*−/−*^ and wild type littermates (Supplementary Fig. 1A,B).

### Effect of PXMP4 deficiency on peroxisome morphology, numbers, and function under standard chow conditions

Inactivation of various peroxisomal proteins has been shown to disrupt peroxisome proliferation, peroxisome morphology or function in many different organisms including mice and humans^1,3–5^. Ultrastructural analysis using electron microscopy showed that peroxisome number and morphology were not visibly altered in the livers of *Pxmp4*^*−/−*^ mice compared to wild type littermates (Supplementary Fig. 2A,B).

**Figure 2:**
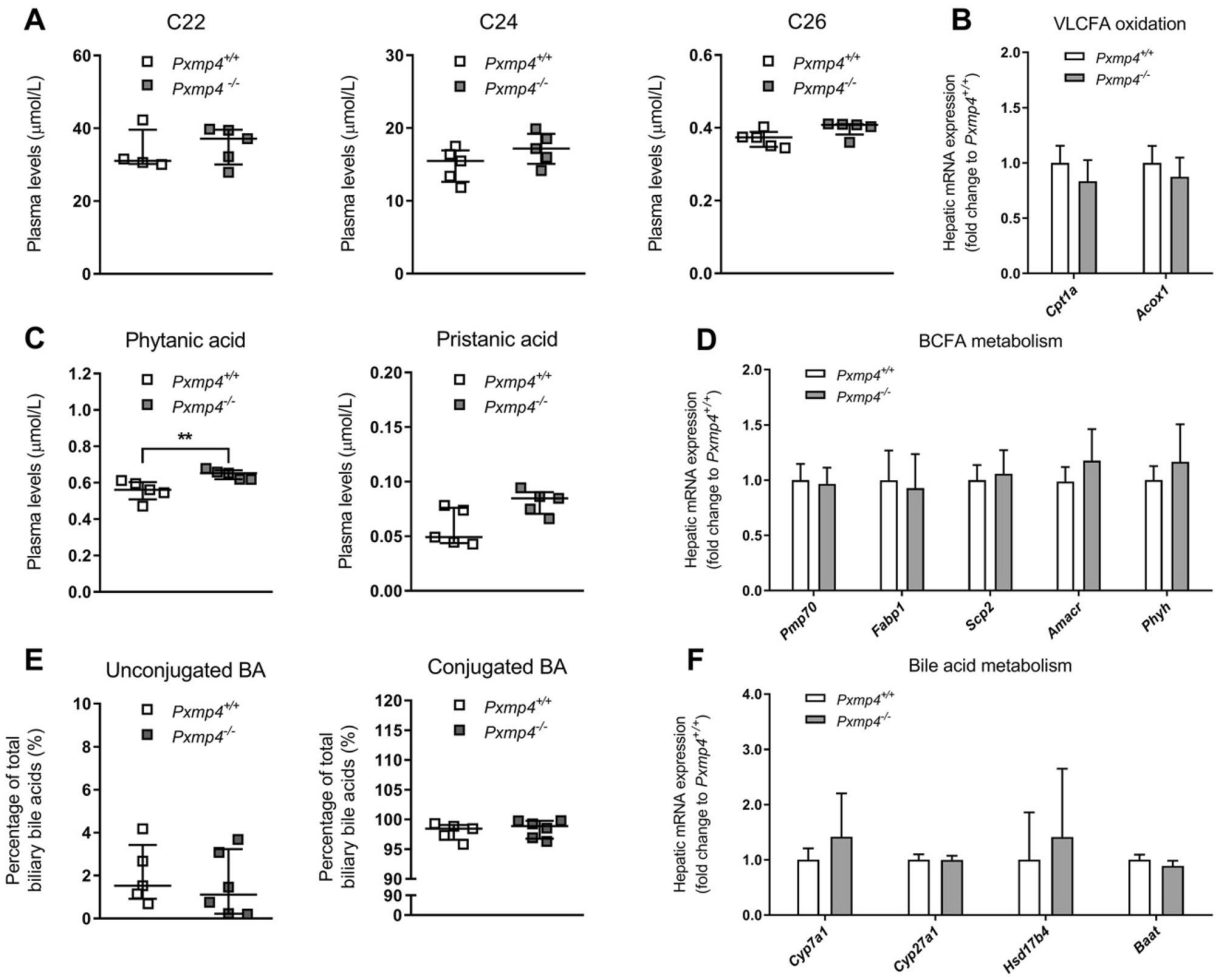
Effect of PXMP4 deficiency on peroxisome function under standard chow conditions. (**A**) Plasma levels of the VLCFAs docosanoic acid (C22), lignoceric acid (C24) and hexacosanoic acid (C26); (**B**) Hepatic expression of genes involved in VLCFA oxidation; (**C**) Plasma levels of the BCFAs phytanic and pristanic acid; (**D**) Hepatic expression of genes involved in metabolism of BCFAs phytanic and pristanic acid; (**E**) Biliary unconjugated and conjugated bile acid concentrations; (**F**) Hepatic expression of genes involved in bile acid metabolism in *Pxmp4*^*−/−*^ mice and wild type littermates (n = 5–6). Scatter plots represent individual data with a median ± IQR and statistical significance was tested by a Mann–Whitney test. Bar plots represent mean ± SD and significance was tested by the non-parametric one-way ANOVA (Kruskal–Wallis) test, followed by Mann–Whitney U tests.

To assess whether PXMP4 deficiency affected peroxisomal α- and β-oxidation of VLCFA and other lipids, we measured VLCFA levels in plasma^14^. In *Pxmp4*^*−/−*^ mice, plasma levels of docosanoic acid (C22), lignoceric acid (C24), and hexacosanoic acid (C26) were not different as compared to wild type littermates (Fig. 2A). Hepatic expression of genes involved in mitochondrial and peroxisomal β-oxidation, respectively, which includes *carnitine palmitoyltransferase 1a* (*Cpt1a*) and *acyl-coenzyme A oxidase 1* (*Acox1*), were not differentially expressed between *Pxmp4*^*−/−*^ mice and wild type littermates (Fig. 2B).

The α-oxidation of phytanic acid and β-oxidation of pristanic acid exclusively occurs in peroxisomes, and various peroxisomal disorders are therefore characterized by increased phytanic and pristanic acid levels^1^. In our study, *Pxmp4*^*−/−*^ animals displayed slightly elevated plasma levels of phytanic (respectively 0.56 vs. 0.65 µmol/L, *p* = 0.008), but not of pristanic acid (respectively 0.05 vs. 0.08 µmol/L, *p* = 0.056) (Fig. 2C) as compared to wild type littermates. Transport and metabolism of phytanic and pristanic acid by the peroxisome involves the concerted action of multiple enzymes as well as several transporters and cofactors, including ATP-binding cassette sub-family D member 3 [ABCD3 or peroxisomal membrane protein 70 (PMP70)], liver fatty acid-binding protein 1 (FABP1 or L-FABP), sterol-carrying protein 2 (SCP2), α-methylacyl-CoA racemase (AMACR) and phytanoyl-CoA 2-hydroxylase (PHYH)^7,30,31^. PXMP4 deficiency, however, did not result in changes in the expression of any of the genes encoding these proteins in the liver (Fig. 2D).

Because hepatic peroxisomes are involved in side-chain shortening of C27-bile acid intermediates and conjugation of C24-bile acids^32^, we analyzed unconjugated and conjugated bile acid species in bile and plasma using LC–MS (Fig. 2E, Supplementary Figs. 3A, 4A, 5A). Biliary and plasma levels of unconjugated, conjugated, or individual BA species were not different between *Pxmp4*^*−/−*^ mice and wild type littermates under standard chow conditions. In line with this, hepatic gene expression of *cholesterol 7 alpha-hydroxylase* (*Cyp7a1*), *sterol 27-hydroxylase* (*Cyp27a1*), *hydroxysteroid (17-beta) dehydrogenase 4* (*Hsd17b4 or D-bifunctional protein* (*Dbp*)), and *bile acid-CoA amino acid N-acyltransferase* (*Baat*) which are all involved in BA metabolism, were also not changed (Fig. 2F). Taken together, our results indicate that PXMP4 deficiency has a minor effect on phytanic acid homeostasis whereas other tested peroxisome functions such as β-oxidation of VLCFAs and BA synthesis including BA conjugation were not affected.

### Effect of PXPM4 deficiency on peroxisome function upon PPARα-stimulation

As loss of PXMP4 did not alter peroxisome function under normal conditions, we next determined the relevance of PXMP4 after stimulating peroxisome activity by enhancing PPARα activity using fenofibrate (FF). Administration of FF (0.2% w/w) for two weeks in the diet resulted in significant decreases in the body weight of both *Pxmp4*^*−/−*^ mice and wild type littermates compared to chow without affecting relative fat mass (Supplementary Fig. 1A,B). As expected, FF led to the increased hepatic expression of *Pmp70* and the PPARα target genes *Cyp4a10, Cyp4a14,* and *acetyl-CoA acyltransferase 1 (Acaa1)* in both genotypes, whereas *Pxmp4* expression was only increased in wild type mice (Supplementary Fig. 1C,F).

**Figure 3:**
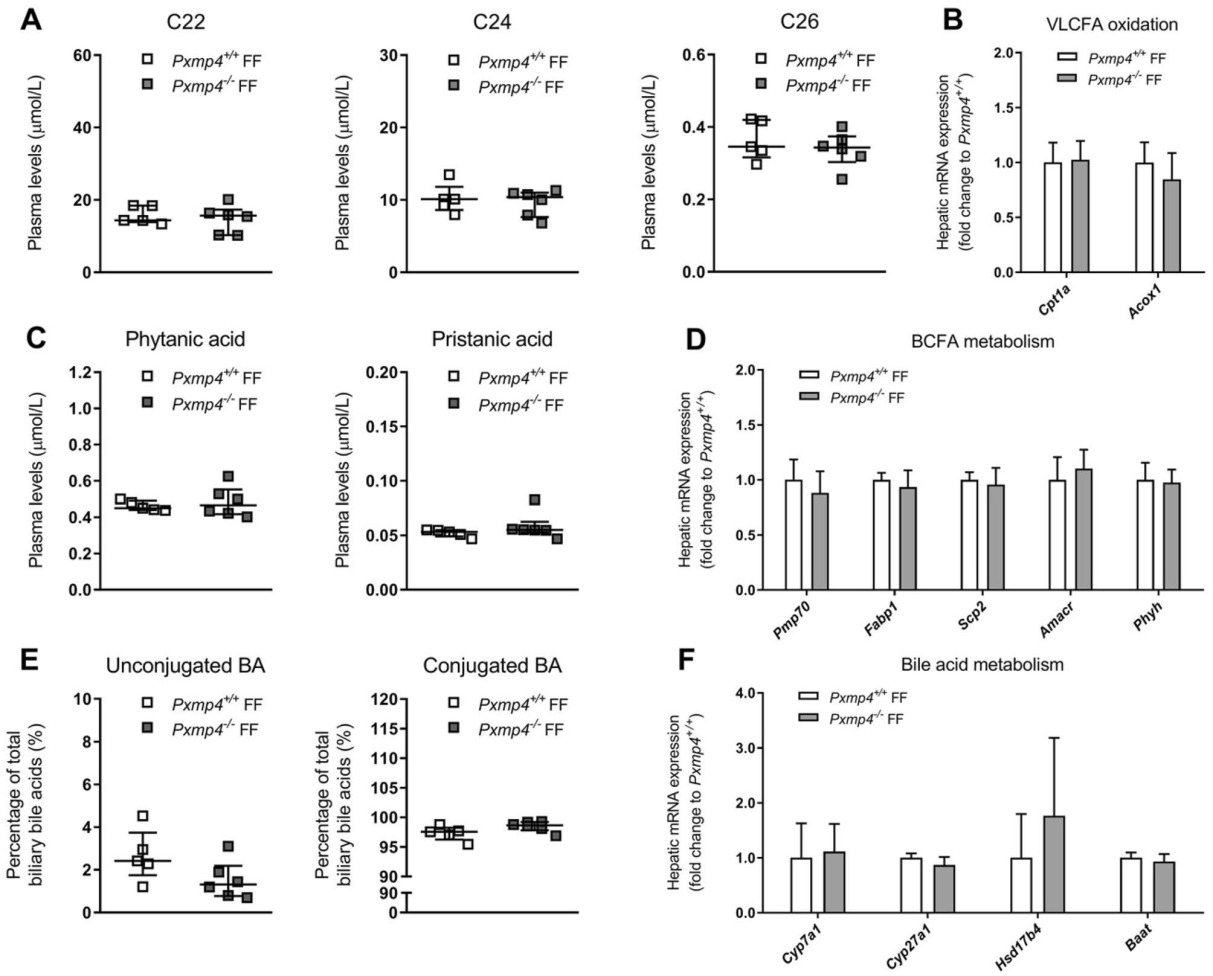
Effect of PXMP4 deficiency on stimulation of peroxisome function by FF. (**A**) Plasma levels of the VLCFAs docosanoic acid (C22), lignoceric acid (C24) and hexacosanoic acid (C26); (**B**) Hepatic expression of genes involved in VLCFA oxidation; (**C**) Plasma levels of phytanic and pristanic acid; (**D**) Hepatic expression of genes involved in metabolism of phytanic and pristanic acid; (**E**) Biliary unconjugated and conjugated bile acid concentrations; (**F**) Hepatic expression of genes involved in bile acid metabolism in *Pxmp4*^*−/−*^ mice and wild type littermates (n = 5–6). Scatter plots represent individual data with a median ± IQR and statistical significance was tested by a Mann–Whitney test. Bar plots represent mean ± SD and significance was tested by the non-parametric one-way ANOVA (Kruskal–Wallis) test, followed by Mann–Whitney U tests.

Plasma levels of VLCFAs or hepatic expression of PPARα target genes *Cpt1a* and *Acox1* were not different between *Pxmp4*^*−/−*^ and wild type littermates after FF administration (Fig. 3A,B). However, FF significantly decreased plasma levels of C22 and C24 in both genotypes compared to chow conditions (Supplementary Fig. 1D). FF decreased plasma levels of C26 only in *Pxmp4*^*−/−*^ mice (0.40 vs. 0.34 µmol/L, *p* = 0.048), but not in wild type mice (0.37 vs. 0.36 µmol/L, *p* = 0.969). Although administration of FF did not change plasma levels of phytanic and pristanic acid between *Pxmp4*^*−/−*^ mice and wild type littermates (Fig. 3C), FF did lower the levels of plasma phytanic and pristanic acid in *Pxmp4*^*−/−*^ mice compared to standard chow conditions (Supplementary Fig. 1E). In wild type mice, FF decreased plasma levels of phytanic acid, but not pristanic acid, compared to standard chow. Hepatic expression of *Pmp70, Fabp1*, *Scp2, Amacr,* and *Phyh* were not altered (Fig. 3D). Administration of FF did not result in different levels of unconjugated, conjugated, or individual BA species in bile or plasma between *Pxmp4*^*−/−*^ mice and wild type littermates (Fig. 3E, Supplementary Figs. 3B, 4B, 5B). Also, the expression of genes involved in bile acid metabolism was not altered (Fig. 3F). Taken together, these results indicate that *Pxmp4*^*−/−*^ mice do not have an overt phenotype under standard chow dietary conditions or after PPARα-dependent stimulation of peroxisomal activity by FF.

### Effect of PXPM4 deficiency on peroxisome function after administration of phytol

**Figure 4:**
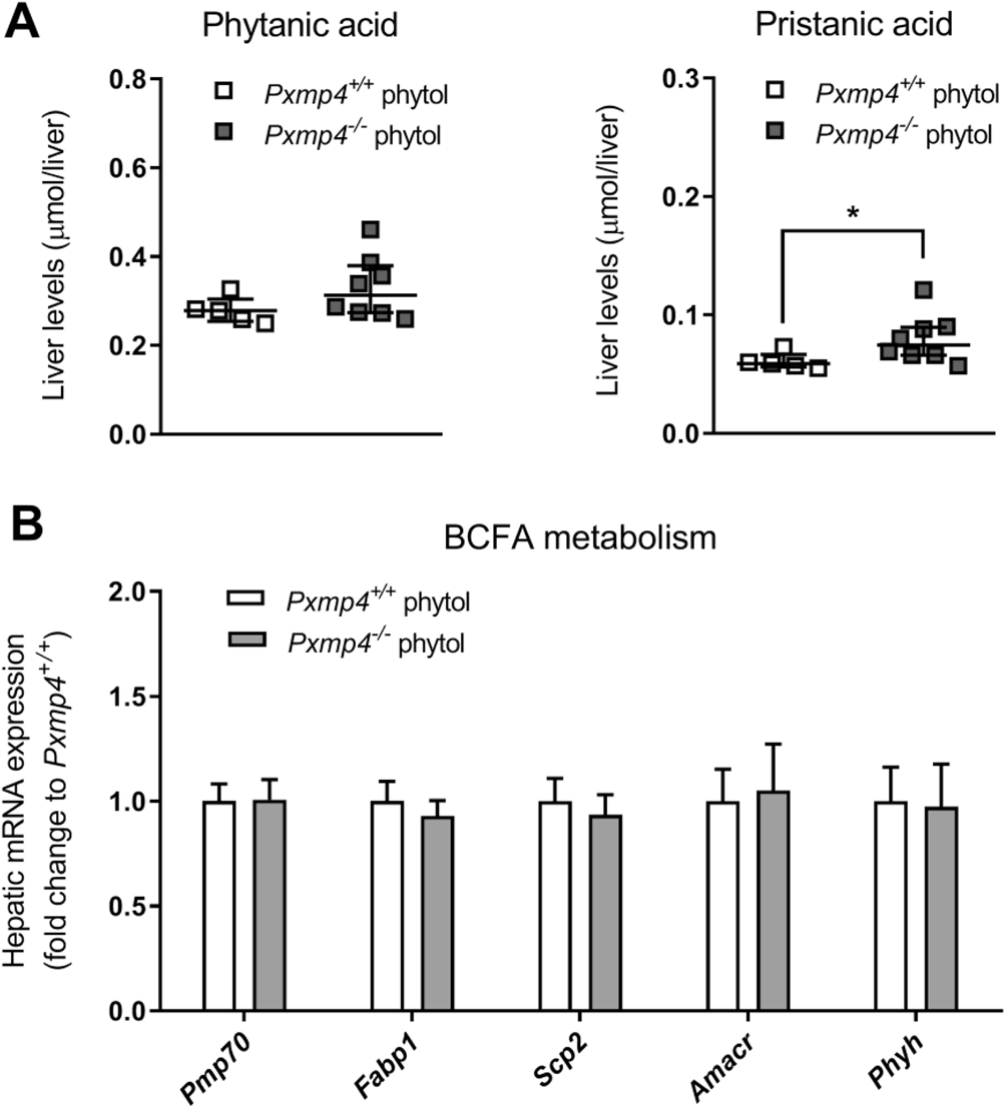
Effect of PXMP4 deficiency on metabolism of phytanic and pristanic acid. (**A**) Hepatic levels of phytanic and pristanic acid. Data is represented as individual data with a median ± IQR and statistical significance was tested by a Mann–Whitney test; (**B**) Hepatic expression of genes involved in metabolism of phytanic and pristanic acid in *Pxmp4*^*−/−*^ mice and wild type littermates (n = 6–8). Data is represented as mean ± SD and significance was tested by the non-parametric one-way ANOVA (Kruskal–Wallis) test, followed by Mann–Whitney U tests**.**

Earlier studies investigating mouse models for peroxisomal disorders have used the chlorophyll metabolite phytol to study peroxisomal α- and β-oxidation pathways^7,31,33–37^. Phytol is converted into the branched-chain fatty acids (BCFAs) phytanic and pristanic acid stepwise through the concerted action of multiple enzymes distributed among different subcellular organelles in the cytosol, endoplasmic reticulum, peroxisome, and mitochondrion^14^. A deficiency in the oxidation of phytol, as in mice deficient in phytanoyl-CoA hydroxylase, can lead to a severe phenotype characterized by the accumulation of phytanic acid with or without elevated pristanic acid, resulting in liver failure, hepatic steatosis, infiltration of inflammatory cells, and peripheral neuropathy^7,31,38^. Because of the mildly elevated levels of phytanic acid in *Pxmp4*^*−/−*^ mice under standard chow-fed conditions, we decided to study if dietary supplementation of phytol in the diet (0.25% w/w) for four weeks could induce a phenotype in *Pxmp4*^*−/−*^ mice^35–37^. Phytol treatment was effective as indicated by increased hepatic expression of *Pmp70* (Supplementary Fig. 6A) and the PPARα target genes *Cyp4a10, Cyp4a14,* and *Acaa1* (Supplementary Fig. 6D) in all mice. Effective phytol treatment was also evidenced by a strong increase in peroxisome number observed in electron microscopic images (from 15 ± 6 to 53 ± 27 peroxisomes/hepatocyte in *Pxmp4*^*−/−*^ mice and 13 ± 5 to 54 ± 19 in wild type littermates) (Supplementary Fig. 2C,D). However, peroxisome numbers were not different between genotypes. Furthermore, except for mildly elevated levels of pristanic acid in *Pxmp4*^*−/−*^ mice, no overt toxicity was observed. Also, upon phytol supplementation, *Pxmp4*^*−/−*^ mice did not show differences in body weight, fat mass, and hepatic levels of phytanic acid compared to wild type littermates (Fig. 4A; Supplementary Fig. 6B,C). Moreover, we did not observe any differences in the expression of genes involved in phytanic or pristanic acid metabolism between *Pxmp4*^*−/−*^ mice and wild type littermates (Fig. 4B).

### Effect of PXPM4 deficiency on the hepatic lipidome

**Figure 5:**
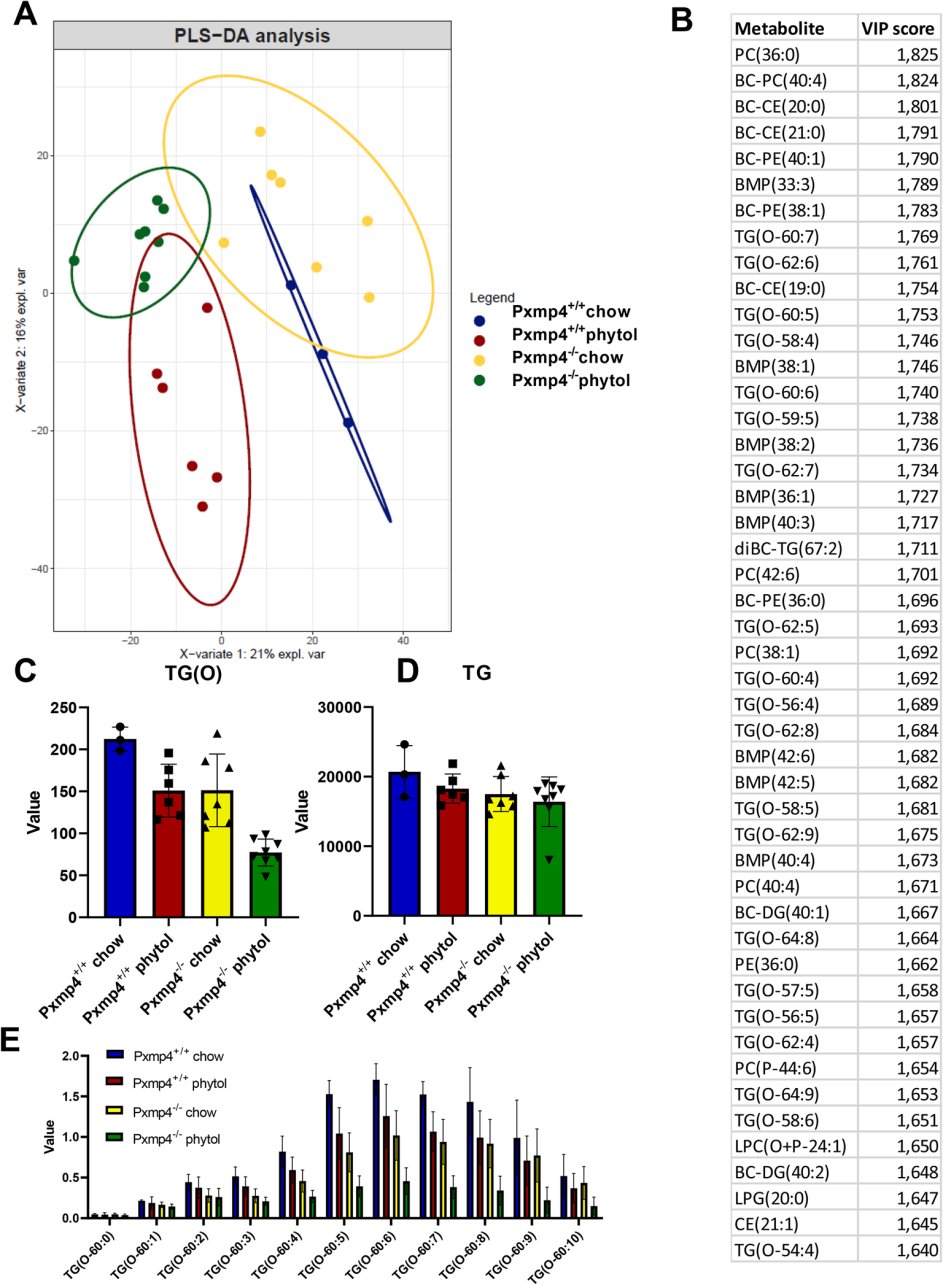
Effect of PXMP4 deficiency and phytol supplementation on the hepatic lipidome. (**A**) Partial Least Square (PLS) regression analysis; (**B**) Most important changed lipid species based on PLS-derived VIP scores; (**C**) Effect of PXMP4 deficiency and phytol supplementation on total hepatic TG levels; (**D**) Effect of PXMP4 deficiency and phytol supplementation on total hepatic TG(O) levels; (**E**) Effect of PXMP4 deficiency and phytol supplementation on the hepatic abundance of TG(O-60) species.

As the loss of PXMP4 did not impair metabolism of VLCFAs or bile acids, we next explored its potential role in lipid metabolism by performing targeted lipidome analysis on the liver of *Pxmp4*^*−/−*^ mice and wild type littermates, with or without phytol supplementation. Partial Least Square (PLS) regression analysis showed that phytol supplementation explained most of the observed variance in the hepatic lipidome (Fig. 5A). The top 50 most changed lipid species based on PLS-derived VIP scores appeared to be strongly enriched in various branched-chain (BC), bismonoacylglycerophosphate (BMP), phosphatidylcholine (PC), and alkyldiacylglycerol (TG(O)) lipid species (Fig. 5B). Further stratification revealed that the increased levels of various branched-chain-, and BMP lipid species were largely explained by phytol supplementation and most likely reflect the incorporation of the branched chain fatty acid phytanic acid and its fatty acid metabolites, after initial conversion from phytol, into these complex lipids (Supplementary Fig. 7). The levels of these BC, PC, and BMP lipids, however, were not different between *Pxmp4*^*−/−*^ mice and wild type littermates (Supplementary Fig. 7) further underscoring that Pxmp4 is not involved in branched-chain fatty acid metabolism.

While the abundance of the majority of the > 1000 analyzed lipids was not substantially affected by PXMP4 deficiency, we observed a moderate decrease in TG[O] in the liver of *Pxmp4*^*−/−*^ mice as compared to wild type littermates, and this lipid species was even further decreased upon phytol treatment (Fig. 5C). In contrast, loss of Pxmp4 or phytol treatment did not affect total hepatic TG levels (Fig. 5D). Closer examination of the effect of phytol on the TG[O] species profile showed that the levels of polyunsaturated TG[O] species were more strongly reduced as compared to the saturated, mono- di and tri-saturated species, which were present at comparable levels (Fig. 5E). On the other hand, species likely containing less unsaturated fatty acids (i.e. C18:2, C18:3, C20:4) were increased by phytol. This fatty acid effect was also present in TG species and occurred in both genotypes although more prominent in *Pxmp4*^*−/−*^ mice (Fig. 5B; Supplementary Fig. 8). There was no clear deficiency of polyunsaturated species in other major classes. Overall, these observations suggest that PXMP4 has a role the metabolism of ether lipids, particularly those containing polyunsaturated fatty acids.

## Discussion

In this study, we investigated the function of the peroxisomal PPARα target PXMP4 using a total body knockout mouse model. While *Pxmp4* deficient mice displayed no obvious defects in VLCFA and bile acid metabolism under standard chow conditions or after stimulation of PPARα, decreased hepatic levels of neutral ether lipids, particularly the ones containing polyunsaturated fatty acids suggest a role for PXMP4 in their metabolism.

Although identified in 1999, the function of PXMP4 has remained unknown^27^. The lack of an overt phenotype in knockout mice indicates that the function of PXMP4 is not essential or redundant under the tested conditions for the indicated metabolic pathways. In contrast to what has been reported for several other single peroxisomal protein deficiencies, no alterations in VLCFA levels or conjugated bile acids were found between *Pxmp4*^*−/−*^ and wild type littermates^5,6^. Based on decreased PXMP4 expression in NOD mice, which have a deficiency in the numbers and function of natural killer T-cells (NKT) cells, Fletcher et al. proposed a role for PXMP4 in the adaptive and innate immune system by regulation of numbers of NKT type 1 (NKT1) cells. NKT1 cells are activated by the antigen-presenting molecule cluster of differentiation 1 (CD1) family, and ligands for CD1d include ether phospholipids, which are synthesized by peroxisomes^39^. Based on these findings, it has been proposed that PXMP4 is involved in ligand availability for CD1d. However, the role of PXMP4 and other peroxisomal proteins in the supply of ligands for NKT1 activation remains unclear^40^. Metabolic processes, including fatty acid oxidation, are aberrant during carcinogenesis, and due to their important metabolic function, a role for peroxisomes in cancer has been suggested. Several peroxisomal proteins such as α-methylacyl-CoA racemase (AMACR) are elevated in various tumour types, including prostate cancer (PCa) and patients with PCa often display increased plasma phytanic acid levels^2,41^. PXMP4 has been implicated in the development of PCa because it was transcriptionally silenced by the epigenetic process of DNA hypermethylation in a human PCa cell line^42,43^. Conversely, overexpression *of Pxmp4* in LNCaP cells and the prostate cancer-3 (PC-3) cell line resulted in a decrease in the number of cancer cells (by − 27% and − 36%, respectively), indicating that *Pxmp4* could function as a tumor suppressor gene^42^. However, the individual roles of many peroxisomal proteins, including PXMP4, in carcinogenesis are not conclusive and therefore remain to be elucidated. We did not investigate the incidence of PCa in our *Pxmp4*^*−/−*^ mouse model because mice are relatively resistant to the development of PCa^44^.

PXMP4 is a target of PPARα and this nuclear receptor can be activated by fibrates as well as by the branched-chain fatty alcohol phytol and its metabolites^45–47^. PPARα activation resulted in decreased plasma VLCFA levels and changes in conjugated bile acids levels, in line with other studies^48–52^. PPARα activation was also supported by changes in hepatic gene expression of PPARα target genes involved in these metabolic pathways both in the knockout mice and wild type littermates and reported in earlier studies^53^.

Despite the lack of an overt phenotype in *Pxmp4*^*−/−*^ mice, increased plasma levels of phytanic acid and hepatic levels of pristanic acid pointed towards an impairment in α-oxidation. Interestingly, the increase in phytanic and pristanic acid in *Pxmp4*^*−/−*^ mice compared to wild type littermates under standard chow disappeared after FF treatment. This was also reported for *Phyh*^*−/−*^ mice that were treated with 0.1% FF for 2 weeks^54^. In that study, co-administration of FF and phytanic acid decreased plasma phytanic acid levels compared to administration to the diet of phytanic acid alone. A possible explanation for this effect is that FF increases the ω-oxidation of phytanic acid in the liver, thereby decreasing levels in plasma.

Based on our finding that *Pxmp4*^*−/−*^ mice have elevated plasma phytanic acid levels under chow conditions, we hypothesized that PXMP4 could be involved in the peroxisomal oxidation of phytol. The branched-chain alcohol phytol is a precursor for phytanic acid but usually is present at low amounts in the standard rodent diet. Therefore, dietary supplementation of phytol has often been used to challenge the peroxisomal metabolism of phytanic acid and subsequently pristanic acid in mice^7,31,33–37^. Phytol mainly accumulates in the liver, where it is first converted in the ER into phytanic acid followed by uptake into the peroxisomes where it is converted into pristanic acid via α-oxidation^55^. In our study, phytol supplementation resulted in increased hepatic levels of phytanic and pristanic acid, and this was slightly more pronounced in *Pxmp4*^*−/−*^ mice compared to wild type littermates. In other mouse models in which phytol administration was used, hepatic levels of phytanic and pristanic acid were higher than in our study, indicating that the breakdown of phytol is not limited by PXMP4 deficiency^30,36,38^. Together, these findings demonstrate that upon activation of PPARα by FF or by phytol, an evident phenotype was still absent in *Pxmp4*^*−/−*^ mice. While our study did not identify a critical role for PXMP4 under standard dietary conditions or after stimulation of PPARα, we can not exclude that PXMP4 is important under other conditions where lipid metabolism is challenged, such as high-fat diet feeding or fasting.

Further analysis of the hepatic lipidome revealed reduced levels of TG(O), particularly species containing polyunsaturated fatty acids, in *Pxmp4*^*−/−*^ mice, and this decrease was more pronounced after phytol treatment. Ether lipids have various biological functions including roles in membrane structure, and emerging studies suggest that they are involved in cell differentiation and signaling pathways^56^. Altered ether lipid production is also associated with several disorders including neurodegenerative diseases, cancer, and metabolic disorders^56^. The exact role, however, of PXMP4 in ether lipid metabolism and its relevance for physiology and disease remains to be further investigated.

The metabolic function of several peroxisomal proteins has not yet been elucidated. Several other studies investigating a single peroxisomal protein-deficient mouse model also reported no (evident) phenotype. Atshaves et al. reported that under chow conditions, both male and female *Scpx* deficient (*Scpx*^*−/−*^) mice had no altered body weight, liver weight, fat mass or lean mass compared to their wild type littermates. However, plasma and liver lipids were decreased in *Scpx*^*−/−*^ mice and regulated in a gender-dependent manner^30^. Peroxisomal membrane protein 34 knockout (*Pmp34*^*−/−*^) animals, either under chow-fed conditions or after clofibrate administration, lacked a significant phenotype compared to control animals^57^. Mice with a deficiency in 2-hydroxyacyl-CoA lyase 1 (HACL1), a key enzyme in α-oxidation of phytanic acid, also displayed no divergent phenotype under chow-fed conditions^38^. The enzyme AMACR is involved in the racemization of the bile acid synthesis C27-intermediates di- and trihydroxycholestanoic acid (DHCA and THCA respectively), as well as racemization of pristanic acid^58,59^. *Amacr*^*−/−*^ mice had increased biliary and serum C27-intermediates and lower C24 bile acids, but no alterations in phytanic or pristanic acid compared to wild type animals under chow-fed conditions^60^. Despite the changes in bile acid metabolism, no clinical phenotype was found in these *Amacr*^*−/−*^ mice^60^. Under phytol-fed conditions, our mice only showed increased phytanic acid levels in the liver, but no other peroxisome functions were affected, and no clinical phenotype was found.

Although various somatic mutations and hypermethylation resulting in the silencing of PXMP4 in humans have been reported for several types of cancer, its role in tumor development, as well as its physiological function, has remained unknown. The gnomAD database v2.11 (gnomad.broadinstitute.org) lists more than 300 single nucleotide variants (SNVs) for human PXMP4, including 21 indels, 7 frameshifts, 11 splice variants and 10 loss/gain function^61^. PXMP4 was predicted as a gene tolerant to variations, with the following constraint metrics: the probability of being loss-of-function (LoF) intolerant (pLI) = 0 and observed/expected (o/e) = 0.63^62^. Further analysis of the common genetic variants in PXPM4 in the UK Biobank (ukbiobank.ac.uk) and FinnGen (finngen.fi/en) general population cohorts did not yield any lipid-related associated traits (data not shown).

Recently it has been shown that PXMP4 and the transmembrane protein 135 (TMEM135; PMP52) are both members of the Tim17 protein family showing 30% homology^63^. TMEM135 has been associated with fatty acid metabolism and indicated as a PPARα target and could be functionally related to PXMP4^64–66^. However, we did not find differences in hepatic *Tmem135* expression between wild type and *Pxmp4*^*−/−*^ mice after administration of chow, FF, or phytol (data not shown). To assess whether redundancy is caused by other peroxisomal proteins, future studies using double knockout of PXMP4 together with homologous proteins such as TMEM135 could give more insight into the metabolic function of PXMP4.

## Materials and methods

### Animals

A whole-body *Pxmp4* knockout mouse model (*Pxmp4*^*−/−*^) was generated by CRISPR/Cas9-mediated gene editing as previously described^67^. Briefly, FVB females were super ovulated by injection with 5 IU Folligonan (0.2 ml i.p.) and 48 h later with 5 IU Chorulon (0.2 mL i.p.). The next day, zygotes were isolated from the infundibulum and injected with 100 ng/µL *Cas9* RNA + 50 ng/µL sgRNA *Pxmp4* (CGCTGCGCTGGCCGTGATAA). Injected zygotes were incubated overnight at 37 °C and transferred to the infundibulum of pseudopregnant females. Offspring carrying targeted mutations resulting in a loss-of-function protein were selected for breeding with wild type C57BL/6J mice. Germline transmission of the mutation was verified and the mice carrying the mutation were backcrossed (N > 7) into the C57BL/6J background. During the experiments, male and female animals were housed individually, kept in a light- and temperature-controlled environment, and had ad libitum access to standard chow (RM1, Special Diets Services, Essex, UK) and drinking water. This study was carried out in compliance with the ARRIVE guidelines. All experimental procedures were approved by the local Ethics Committee for Animal Experiments of the University of Groningen. Experiments were performed in accordance with relevant guidelines and regulations (including laboratory and biosafety regulations and Directive 2010/63/EU).

### Animal procedures

General characterization of male and female wild type and *Pxmp4*^*−/−*^ mice did not show differences in plasma lipid levels, BAs levels, and the branched-chain fatty acids (BCFAs) phytanic and pristanic acid (data not shown). To investigate the function of PXMP4, male *Pxmp4*^*−/−*^ mice and wild type littermates of 20–24 weeks old were studied under three different experimental conditions: standard chow, PPARα-activation by dietary fenofibrate (FF) administration, and overloading of the α-oxidation pathway by dietary phytol administration. Before every experiment, basal parameters, including food intake (24 h) and body composition, were measured. The body composition (total fat mass, lean mass, and fluid) was determined by nuclear magnetic resonance using the Bruker MiniSpec LF110 BCA-Analyzer (Bruker Optics Inc, Billerica, MA, USA). To determine basal blood parameters, blood was collected from the hind paw after 3 h of fasting. Food intake was monitored two times and bodyweight 2–3 times per week during the experimental period. Animals under chow conditions (n = 5–6) were followed for 2 weeks. For the PPARα-activation study, animals (n = 5–6) were treated with a diet containing 0.2% w/w FF (F6020-5G, Sigma-Aldrich, St Louis, MO, USA) for two weeks. Phytol is a precursor of phytanic acid and is often used to overload the α-oxidation pathway in peroxisomes. Accumulation of phytol and its metabolites phytanic and pristanic acid is toxic and can lead to severe damage to the liver. Because administration of a diet containing 0.5% w/w phytol has been shown to induce hepatotoxicity in mice^35^, we administered a diet containing 0.25% w/w phytol (> 97% sum of isomers, obtained as viscous liquid from Sigma-Aldrich, St Louis, MO, USA) for 4 weeks to *Pxmp4*^*−/−*^ mice and wild type littermates (n = 6–8). The body composition of animals of the chow and FF experiment was monitored at the end of the experimental period. After 2 weeks of control chow diet or FF, or 4 weeks of phytol administration, animals were weighed in the morning, and food was taken away 3 h before gall bladder cannulation as described previously^68^. Briefly, mice were anaesthetized by intraperitoneal injection using a mixture of Hypnorm (fentanyl/fluanisone; 1 mL/kg) and diazepam (10 mg/kg). Bile was collected for 20 min, and bile flow was determined gravimetrically (1 g = 1 mL bile secretion). Terminal blood was obtained through cardiac puncture and stored at − 20 °C. The liver was harvested, flushed with ice-cold PBS and snap-frozen in liquid nitrogen before storage at − 80 °C.

### Electron microscopy

Electron microscopy was performed using standard protocols essentially the same as previously described^69^. In the current study, primary fixation was in 2% glutaraldehyde and 2% paraformaldehyde in 0.1 M Cacodylate buffer (pH 7.4). Samples were stored at 4 °C until further processing. Tissue was osmicated prior to embedding with 1.5% osmium tetroxide/potassium ferrocyanide. Ultrathin (80 nm) sections were placed on single slot (2 × 1 mm) cupper grids and contrasted with Neodymium^70^. Acquisition was on a Zeiss Supra55 ATLAS following procedures described in Ref.^69^. Peroxisome numbers were counted in centrally cross-sectioned hepatocytes (according to a prominent nucleus).

### Gene expression analysis

Snap-frozen livers were crushed using liquid nitrogen, and total RNA was isolated using TRI-reagent (Sigma-Aldrich, St Louis, MO, USA). RNA concentrations were quantified by NanoDrop (NanoDrop Technologies, Wilmington, DE, USA) and subsequently 1 µg used for cDNA synthesis (M-MLV reverse transcriptase, Thermo-Fisher Scientific, Waltham, USA). Real-time quantitative polymerase chain reaction (qPCR) was performed on QuantStudio 7 Flex machine (Applied Biosystems, Thermo Fisher, Darmstadt, Germany) using Fast Advance Taqman Mastermix (Applied Biosystems, Foster City, CA, USA) or Fast Start SYBR Green (Roche, Mannheim, Germany). Taqman and SYBR Green primer sequences used in this study are listed in Tables 1 and 2. Gene expression levels were normalized using *cyclophilin* (Taqman) and *TATA-Box binding protein* (*Tbp*) (SYBR Green) as housekeeping genes (HKG).

**Table 1:** Taqman primer sequences.

Gene	Forward sequence 5′–3′	Reverse sequence 3′–5′	Probe sequence
*Acox1*	GCC ACG GAA CTC ATC TTC GA	CCA GGC CAC CAC TTA ATG GA	CCA CTG CCA CAT ATG ACC CCA AGA CCC
*Baat*	TGT AGA GTT TCT CCT GAG ACA TCC TAA	GTC CAA TCT CTG CTC CAA TGC	CCT CGG CCC AGG TGT TGG CA
*Cpt1a*	CTC AGT GGG AGC GAC TCT TCA	GGC CTC TGT GGT ACA CGA CAA	CCT GGG GAG GAG ACA GAC ACC ATC CAA C
*Cyclophilin*	CAG ATC GAG GGA TCG ATT CAG	TCA CCA CTT GAC ACC CTC ATT C	CTC CTC CAC ATT GGA GAC AAG AGA TGC A
*Cyp27a1*	GCC TTG CAC AAG GAA GTG ACT	CGC AGG GTC TCC TTA ATC ACA	CCC TTC GGG AAG GTG CCC CAG
*Cyp7a1*	CAG GGA GAT GCT CTG TGT TCA	AGG CAT ACA TCC CTT CCG TGA	TGC AAA ACC TCC AAT CTG TCA TGA GAC CTC C
*Dbp (Hsd17b4)*	GAG GAA CAG AAG GAT GAG AAG TAC TG	TGG TTC TCC TTG AGT CTT CTT GC	CTC GAC CTC TTG GCT GCT TCA TTG TTC
*Fabp1*	GAA CTT CTC CGG CAA GTA CCA A	TGT CCT TCC CTT TCT GGA TGA G	CCA TTC ATG AAG GCA ATA GGT CTG CCC
*Pmp70*	CTG GTG CTG GAG AAA TCA TCA AT	CCA GAT CGA ACT TCA AAA CTA AGG T	TGA TCA TGT TCC TTT AGC AAC ACC AAA TGG
*Scp2*	GTG GCT CTG CAG CAC AAT CTA	CTG AAG GAG CTG GCA GCT T	CAA CCA CAG CTC CTC CGA GGC C

**Table 2:** SYBR Green primer sequences.

Gene	Forward sequence (5′–3′)	Reverse sequence (3′–5′)
*Acaa1*	ACA TCT CCG TGG GCA ATG TT	CTC AGA AAT TGG GCG ATG CG
*Amacr*	GAG AAT TTT CTG GCC CGA GG	AGT TTC TCC ATG ACA CCG CA
*Cyp4a10*	CTG GGG CGA TCA GAT CCA AA	TGG GGT TAG CAT CCT CCT GT
*Cyp4a14*	ACG AGC ACA CAG ATG GAG TG	TCT TCT TCC TGG CCT TCT GC
*Phyh*	TAC TGC CTT CTC CCC GAG AT	CGG GAT GTC TTC TTG CCA AC
*Pxmp4*	CGC TGG CCG TGA TAA AGG	GAG AGT GGA TGT ACG TGG CT
*Tbp*	TTC ACC AAT GAC TCC TAT GAC C	CAA GTT TAC AGC CAA GAT TCA CG

### Lipidomics

Lipidomics analysis was performed by the Core Facility Metabolomics of the Amsterdam UMC, as described^71^. The HPLC system consisted of an Ultimate 3000 binary HPLC pump, a vacuum degasser, a column temperature controller, and an auto sampler (Thermo Scientic, Waltham, MA, USA). The column temperature was maintained at 25 °C. The lipid extract was injected onto a "normal phase column" LiChrospher 2 × 250-mm silica-60 column, 5 μm particle diameter (Merck, Darmstadt, Germany) and a "reverse phase column" Acquity UPLC HSS T3, 1.8 μm particle diameter (Waters, Milford Massachusetts, USA). A Q Exactive Plus Orbitrap (Thermo Scientic) mass spectrometer was used in the negative and positive electrospray ionization mode. In both ionization modes, mass spectra of the lipid species were obtained by continuous scanning from *m/z* 150 to *m/z* 2000 with a resolution of 280,000 full width at half maximum (FWHM).

An in-house developed pipeline, written in the R programming language, was used for data processing. The RAW data files were converted to mzXML using MSconvert^72^ in centroided mode. Peak finding and peak group finding was done using the R package XCMS, with minor modifications to some functions for a better representation of the Q Exactive data. Annotation of the peaks was done based on an in-house database containing all possible (phospho)lipid species. Each combination of column (normal phase or reverse phase) and scan mode (positive or negative) was processed separately; after normalization, separate peak group lists were combined into two resulting lists, which were used for statistical analysis. Lipidomics data were analysed using Partial Least Square (PLS) regression analysis and PLC-derived Variable Importance in Projection (VIP) scores were used to identify the most important changed lipid species in the dataset.

### Targeted proteomics

Livers from the mice were snap-frozen using liquid nitrogen and mechanically crushed in liquid nitrogen. A 10% liver homogenate was made using tissue powder together with NP-40 lysis buffer (400 mM NaCl, 1:1000 v/v NP40 (Igepal-640), 10 mM TRIS pH8, 1 mM EDTA pH8). Targeted proteomics was used to quantify PXMP4 in homogenized liver tissue via isotopically labeled peptide standards (containing 13C-labeled lysine), derived from synthetic protein concatamers (QconCAT technology, PolyQuant GmbH Germany) using the workflow previously described^73^. Briefly, in-gel tryptic digestion (1:100 g/g sequencing grade modified trypsin V5111, Promega) was performed on homogenized liver tissues containing 50 μg total protein after reduction with 10 mM dithiothreitol and alkylation with 55 mM iodoacetamide proteins, followed by solid phase extraction (SPE C18-Aq 50 mg/1 mL, Gracepure) for sample cleanup. Detection and quantification of PXMP4 were done targeting the peptide NLACFVFAYK within PXMP4. The isotopically labeled peptide standard was added at a concentration of 0.5 ng concatemer per μg total protein to the liver lysates. These peptides were targeted and analyzed by a triple quadrupole mass spectrometer (MS) equipped with a nano-electrospray ion source (TSQ Vantage, Thermo Scientific). Chromatographic separation was performed by liquid chromatography on a nano-UHPLC system (Ultimate UHPLC focused, Dionex) using a nano-column (Acclaim PepMap100 C18, 75 µm × 500 mm 2 µm, 100 Å) with a linear gradient from 3 to 60% v/v acetonitrile plus 0.1% v/v formic acid in 110 min at a flowrate of 200 nL/min. The MS traces were manually curated using the Skyline software before the integration of the peak areas for quantification^74^. The figures were created using Skyline.

### Biochemical analyses

Determination of plasma VLCFA and the BCFAs phytanic and pristanic acid was performed using gas chromatography combined with mass spectrometry (GC–MS) as described^75^. Individual bile acid species (unconjugated and taurine or glycine conjugated) in bile and plasma were determined using liquid chromatography-mass spectrometry (LC–MS) as described^68^. Total bile acid concentrations were calculated as the sum of the individual bile acid species. The levels of (un)conjugated bile acids were calculated using the percentage of the total sum of bile acids in bile and plasma.

### Statistics

The software GraphPad Prism 8.1 (GraphPad Software, La Jolla, CA, USA) (https://www.graphpad.com/scientific-software/prism/) was used for statistical analyses. Unless stated otherwise, graphs are presented as a scatter dot plot representing individual values with a median ± interquartile range (IQR). Gene expression bars are given as bar plots representing the mean ± standard deviation (SD). Statistical significance between two groups was tested by a non-parametric Mann–Whitney test, and in case of more than two groups by the non-parametric one-way ANOVA (Kruskal–Wallis) test, followed by Mann–Whitney U tests to assess statistical significance between experimental groups. Statistical significance is indicated as *p < 0.05, **p < 0.01, ***p < 0.001 and ****p < 0.0001.

## Supplementary Information

Supplementary Figures.

## Abbreviations

ABCD3: ATP-binding cassette sub-family D member 3
ACOX1: Acyl-coenzyme A oxidase 1
AMACR: Alpha-methylacyl-CoA racemase
BAAT: Bile acid-CoA amino acid *N*-acyltransferase
BA: Bile acid
BCFA: Branched-chain fatty acid
CD1: Cluster of differentiation 1
CPT1A: Carnitine palmitoyltransferase 1a
CYP27A1: Sterol 27-hydroxylase
CYP7A1: Cholesterol 7 alpha-hydroxylase
FABP1: Fatty acid-binding protein 1
FF: Fenofibrate
GC–MS: Gas-chromatography–mass spectrometry
HACL1: 2-Hydroxyacyl-CoA lyase
HKG: Housekeeping gene
HSD17B4: Hydroxysteroid (17-beta) dehydrogenase 4
LC–MS: Liquid chromatography–mass spectrometry
NKT: Natural killer T-cell
PC-3: Prostate cancer-3
PCa: Prostate cancer
PHYH: Phytanoyl-CoA 2-hydroxylase
PMP34: Peroxisomal membrane protein 34
PMP70: Peroxisomal membrane protein 70
PPAR: Peroxisome proliferator-activated receptors
PPARα: Peroxisome proliferator-activated receptor alpha
PXMP4: Peroxisomal membrane protein 4
SCP2: Sterol-carrying protein 2
TBP: TATA-box binding protein
TMEM135: Transmembrane protein 135
VLCFA: Very long-chain fatty acid
