# Supplementary material for: Mice with a deficiency in Peroxisomal Membrane Protein 4 (PXMP4) display mild changes in hepatic lipid metabolism
Source: Sci Rep. 2022 Feb 15;12:2512. doi: 10.1038/s41598-022-06479-y (PMC8847483; doi:10.1038/s41598-022-06479-y)
Supplement: Supplementary file 1 — Supplementary Figures. [file 41598_2022_6479_MOESM1_ESM.docx]

**Mice with a deficiency in Peroxisomal Membrane Protein 4 (PXMP4) display mild changes in hepatic lipid metabolism**

Maaike Blankestijn^a^, Vincent W. Bloks^a^, Dicky Struik^a^, Nicolette Huijkman^a,b^,

Niels Kloosterhuis^a,b^, Justina C. Wolters^a^, Ronald J.A. Wanders^c^, Frédéric M. Vaz^c,d,e^ Markus Islinger^f^, Folkert Kuipers^a^, Bart van de Sluis^a,b^, Albert K. Groen^a,g^,

Henkjan J. Verkade^a^ and Johan W. Jonker^a*^

*^a^ Department of Pediatrics, University of Groningen, University Medical Center Groningen, Groningen, The Netherlands.*

*^b^ iPSC/CRISPR Center Groningen, University of Groningen, University Medical Center Groningen, Groningen, The Netherlands.*

*^c^ Laboratory of Genetic Metabolic Diseases, Amsterdam UMC, University of Amsterdam, Department of Clinical Chemistry, Amsterdam Gastroenterology Endocrinology Metabolism, Amsterdam, The Netherlands.*

*^d^ Department of Pediatrics, Emma Children's Hospital, Amsterdam UMC, University of Amsterdam, Amsterdam, The Netherlands.*

*^e^ Core Facility Metabolomics, Amsterdam UMC, University of Amsterdam, Amsterdam, The Netherlands.*

*^f^Institute of Neuroanatomy, Mannheim Center for Translational Neuroscience, Medical Faculty Mannheim, University of Heidelberg, Mannheim, Germany.*

*^g^ Laboratory of Experimental Vascular Medicine, University of Amsterdam, Academic Medical Center, Amsterdam, The Netherlands.*

**Corresponding author: Department of Pediatrics, University of Groningen, University Medical Center Groningen, Groningen, The Netherlands. Email address:* [*j.w.jonker@umcg.nl*](mailto:j.w.jonker@umcg.nl)

## Supplementary figures


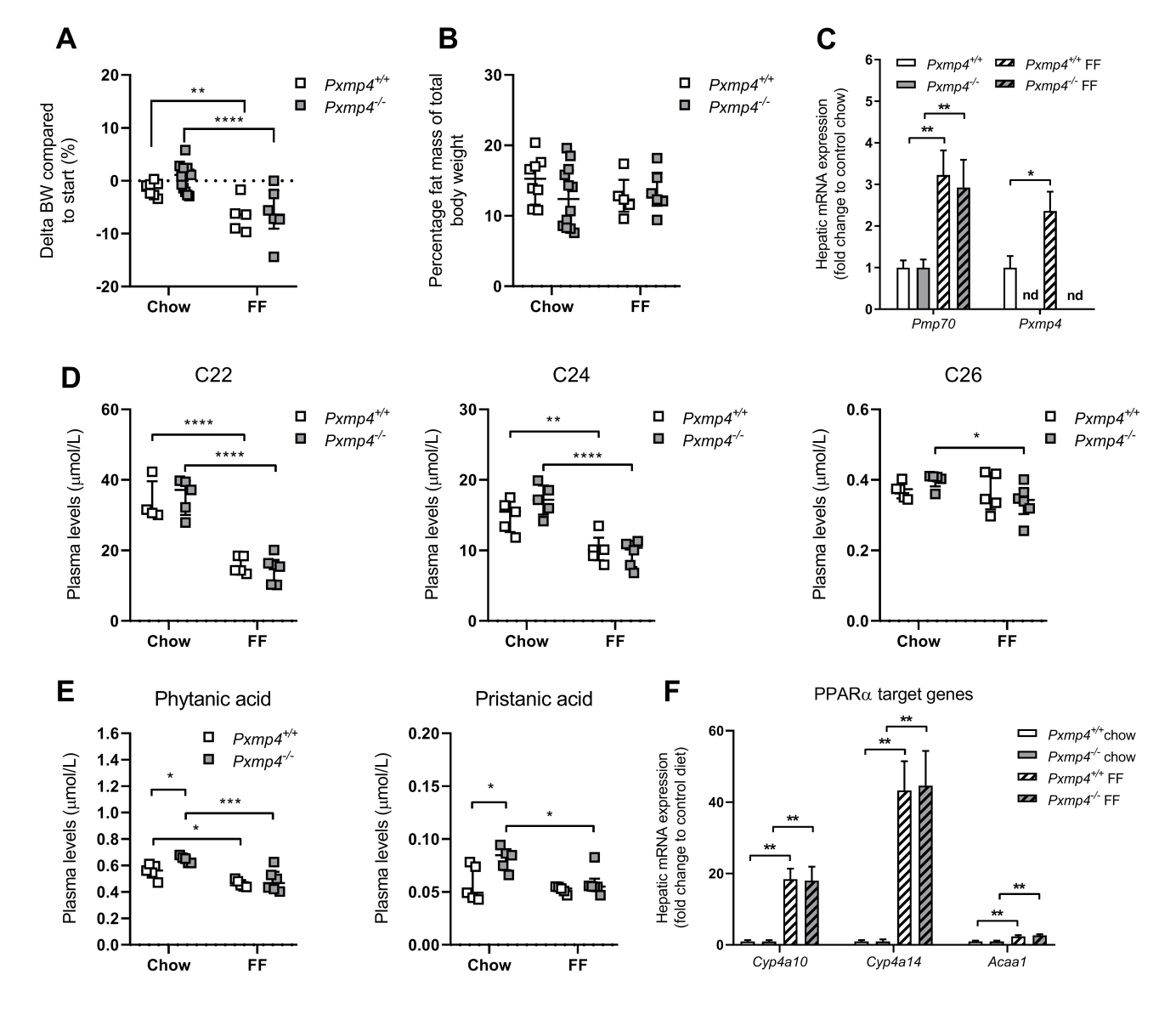


**Supplementary Figure 1. Effect of fenofibrate (FF) on peroxisomal function. (A)** Change in body weight from the start of the experimental period to the end, represented as a percentage (n=5-13); **(B)** Fat mass after feeding with regular chow or FF (n=5-13); **(C)** Hepatic expression of peroxisomal genes after feeding with regular chow or FF (n=5-6); **(D)** Plasma levels of the VLCFAs docosanoic acid (C22), lignoceric acid (C24) and hexacosanoic acid (C26) after administration of chow or FF (n=5-6); **(E)** Plasma levels of the branched-chain fatty acids (n=5-6) after administration of chow or FF in Pxmp4^-/-^ mice and wild type littermates.

**Supplementary Figure 2. Electron microscopic images of livers of** ***Pxmp4^-/-^* mice and wild type littermates under standard chow conditions and after phytol supplementation. (A)** Wild type chow;  **(B)** *Pxmp4^-/-^* chow; **(C)** Wild type phytol**; (D)** *Pxmp4^-/-^* phytol. Peroxisomes are labelled as PO. Glycogen (dark rosettes) specific for hepatic parenchyma was present and visible in many cells. Scale bar: 1μm.


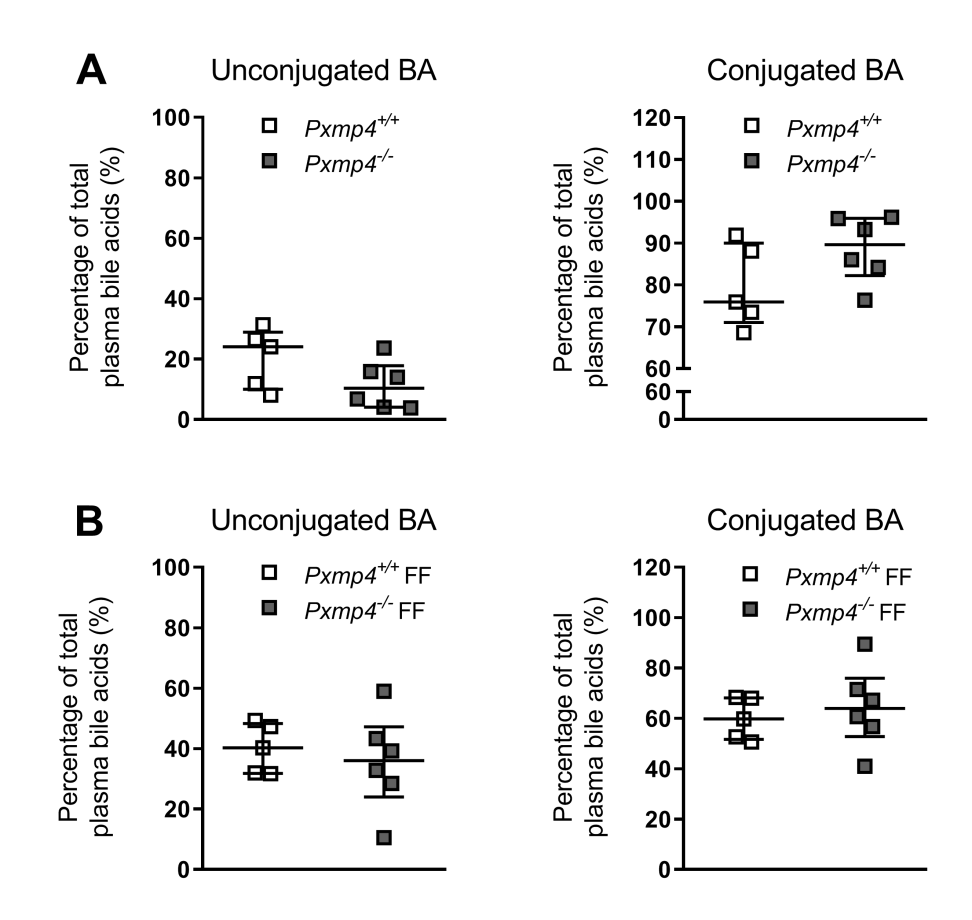


**Supplementary Figure 3.** **Plasma unconjugated and conjugated bile acid concentrations in Pxmp4^-/-^ and wild type littermates** under standard chow conditions **(A)** or after 2 weeks of FF administration **(B).** Number of animals (n=5-6).


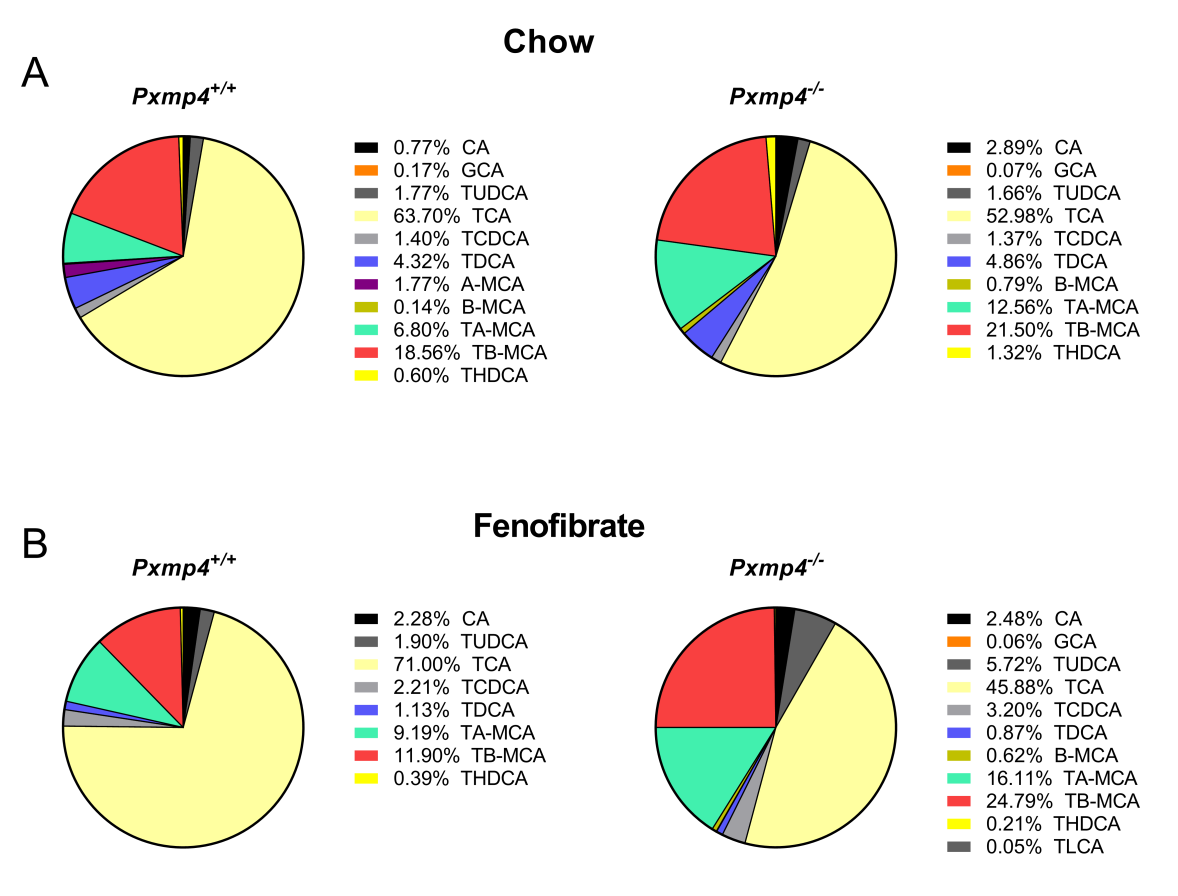


**Supplementary Figure 4.** **Individual bile acid species in bile under standard chow conditions (A) or 2 weeks of FF administration (B).** Number of animals (n=5-6).


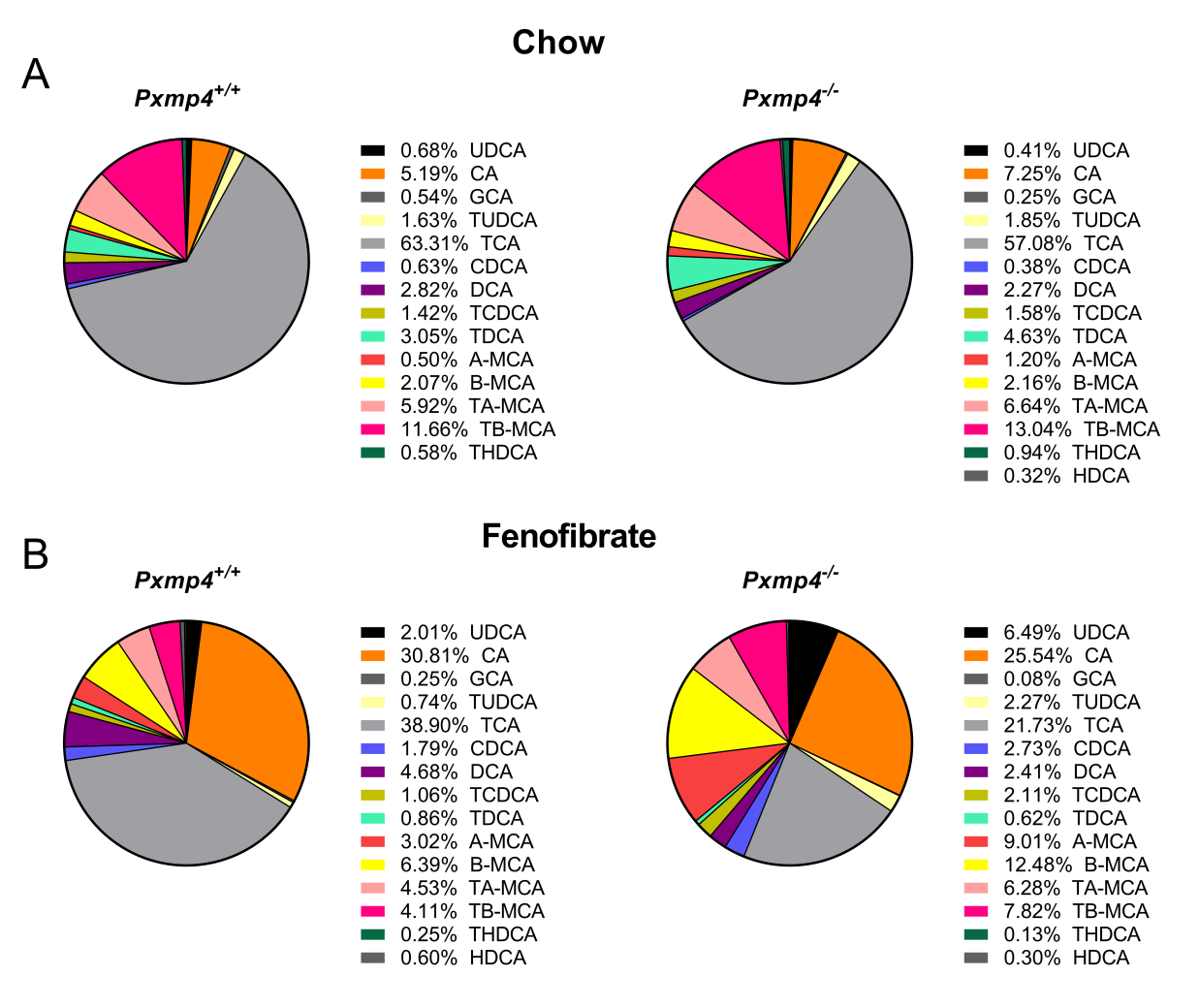


**Supplementary Figure 5.** **Individual bile acid species in plasma** under standard chow conditions **(A)** or after 2 weeks of FF administration **(B).** Number of animals (n=5-6).


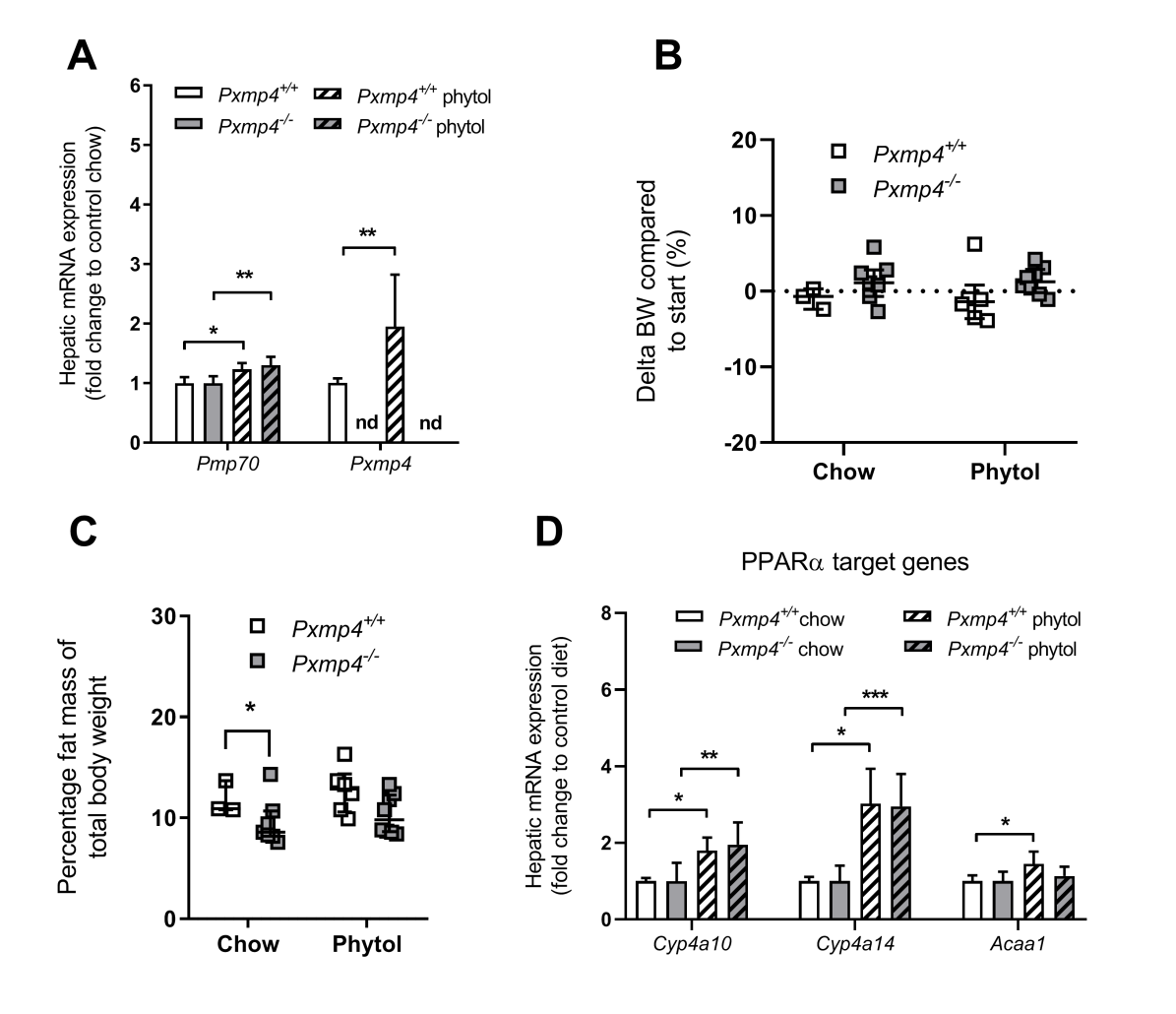


**Supplementary Figure 6. Effect of phytol treatment on peroxisomal function. (A)** Hepatic gene expression of peroxisomal genes after 4 weeks of phytol treatment (n=5-6); **(B)** The difference in body weight (BW) from the end of the experimental period compared to the start of the experiment is given as a percentage; **(C)** Fat mass as a percentage of total body weight after administration of chow or phytol in Pxmp4^-/-^ mice and wild type littermates. Number of animals (n=3-8).

**Supplementary Figure 7. Liver lipidome analysis of *Pxmp4^-/-^* mice and wild type littermates under standard chow conditions and after phytol supplementation. (A)** branched chain phosphatidylcholine 40:4 (BC-PC(40:4)); **(B)** branched-chain cholesteryl ester 20:0 (BC-CE(20:0)); **(C)** bismonoacylglycerophosphate 38:1 (BMP(38:1)); **(D)** bismonoacylglycerophosphate 38:2 (BMP(38:2)); **(E)** phosphatidylcholine 36:0 (PC(36:0)); **(F)** phosphatidylcholine 40:0 (PC(40:0)).

**A**

**
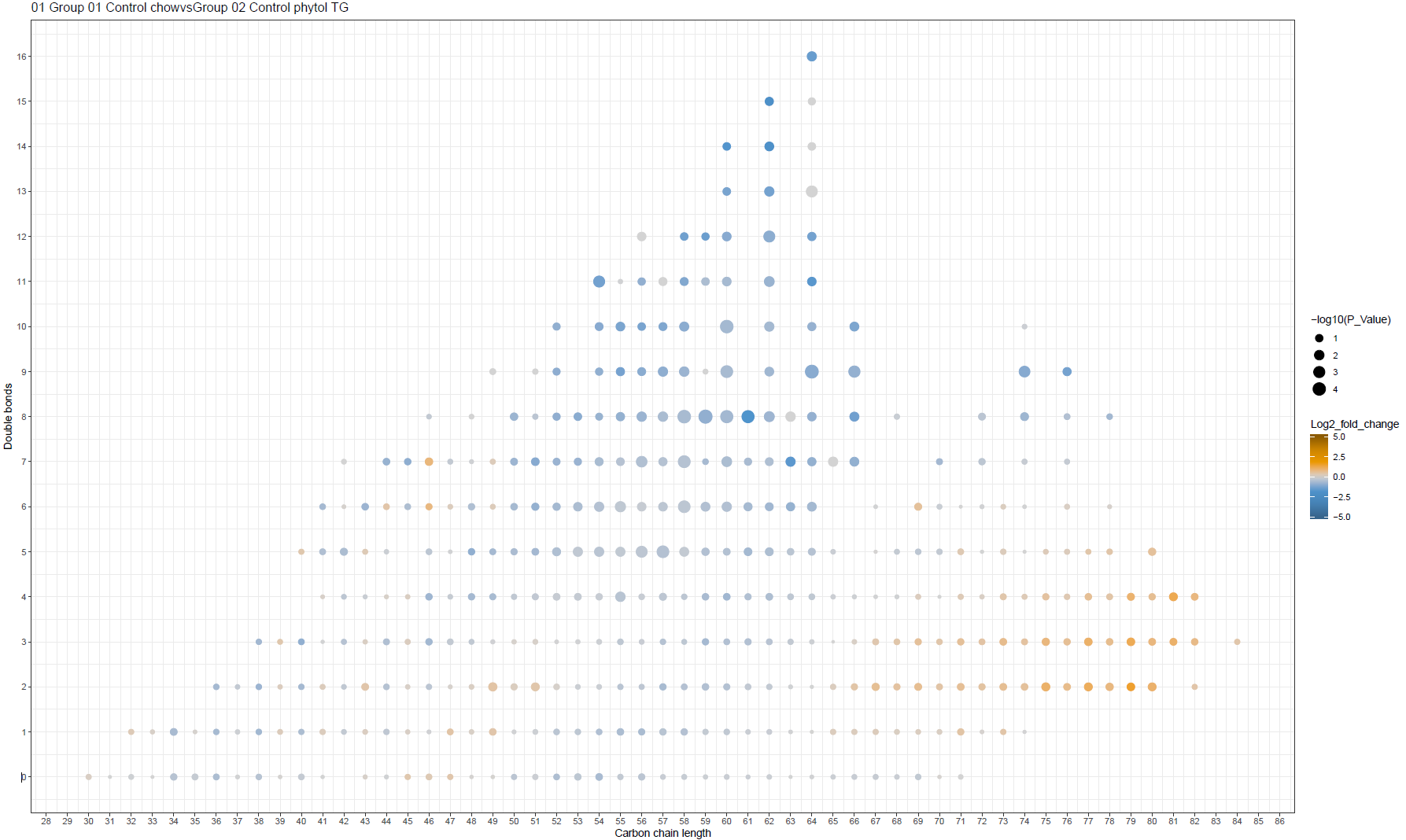
**

B

**
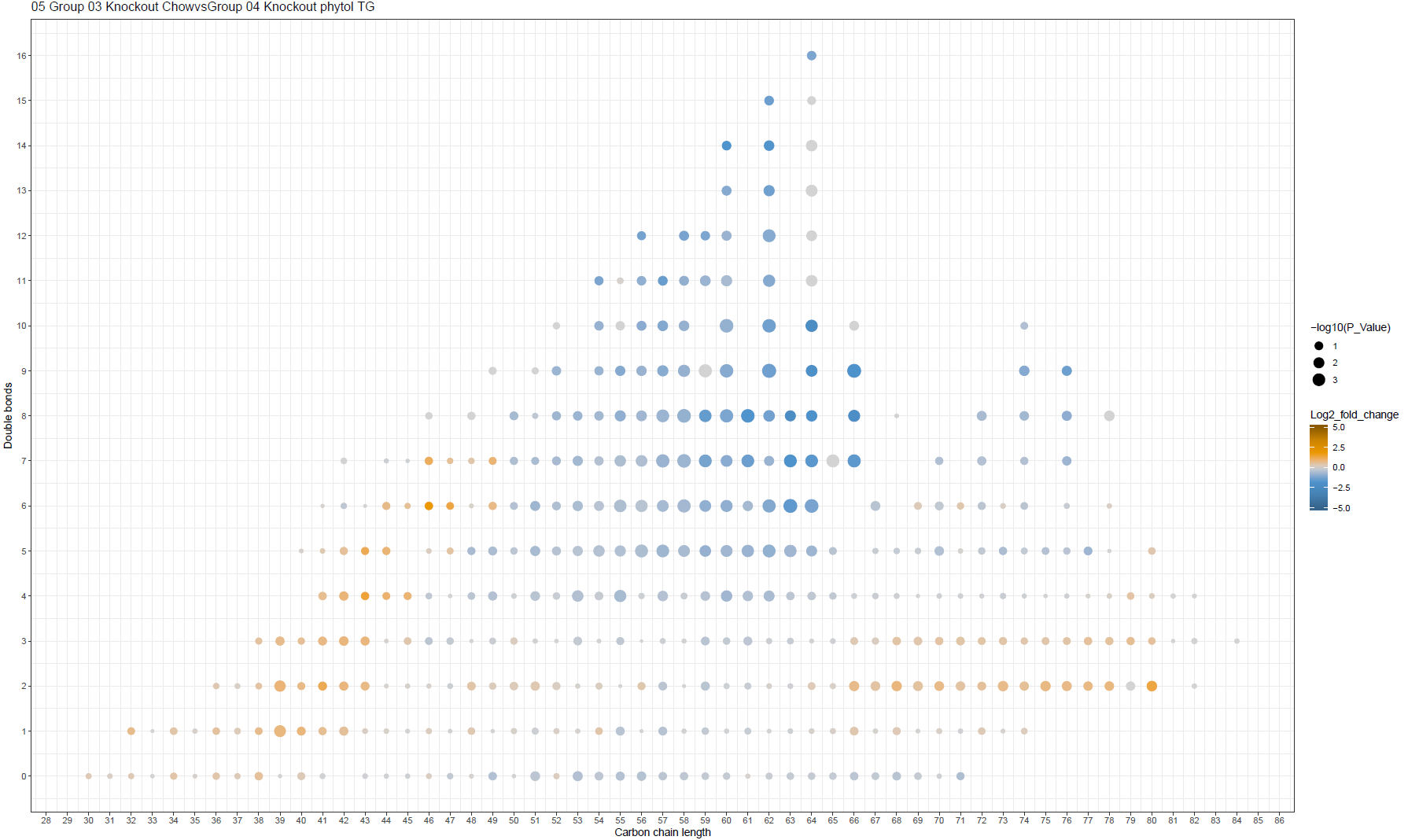
**

**Supplementary Figure 8. Effect of phytol on the fatty acid composition of hepatic TGs** in **A)** wild type versus wild type + phytol and **B)** *Pxmp4^-/-^* mice versus *Pxmp4^-/-^* mice + phytol. Data show the sum of the double bonds in fatty acid side chain(s) (y-axis) versus the sum of the carbon atoms in fatty acid side chain(s) (x-axis). Size of the dots indicate significance (-log10 *p* value) and colors indicate the fold (log2) increase (red) or decrease (blue).
